# Outbreak of enterovirus D68 of the new B3 lineage in Stockholm, Sweden, August to September 2016

**DOI:** 10.2807/1560-7917.ES.2016.21.46.30403

**Published:** 2016-11-17

**Authors:** Robert Dyrdak, Malin Grabbe, Berit Hammas, Jonas Ekwall, Karin E Hansson, Joachim Luthander, Pontus Naucler, Henrik Reinius, Maria Rotzén-Östlund, Jan Albert

**Affiliations:** 1 Department of Clinical Microbiology, Karolinska University Hospital, Stockholm, Sweden; 2 Department of Microbiology, Tumor and Cell Biology, Karolinska Institutet, Stockholm, Sweden; 3 Astrid Lindgren Children’s Hospital, Karolinska University Hospital, Stockholm, Sweden; 4 Department of Infectious Diseases, Södersjukhuset, Stockholm, Sweden; 5 Pediatric Infectious Disease Unit, Astrid Lindgren Children’s Hospital, Karolinska University Hospital, Stockholm, Sweden; 6 Department of Women’s and Children’s Health, Karolinska Institute, Stockholm Sweden; 7 Unit of Infectious Diseases, Department of Medicine Solna, Karolinska Institutet, Stockholm, Sweden; 8 Department of Infectious Diseases, Karolinska University Hospital, Stockholm, Sweden; 9 Department of Anesthesiology and Intensive Care, Akademiska University Hospital, Uppsala, Sweden; 10 Department of Surgical Sciences, Uppsala University, Uppsala, Sweden

**Keywords:** outbreak, enterovirus D68, respiratory infection, acute flaccid myelitis, VP4/VP2 sequencing

## Abstract

We report an enterovirus D68 (EV-D68) outbreak in Stockholm Sweden in 2016. Between 22 August and 25 September EV-D68 was detected in 74/495 respiratory samples analysed at the Karolinska University Hospital. During the peak week, 30/91 (33%) samples were EV-D68 positive. Viral protein (VP)P4/VP2 sequencing revealed that cases were caused by B3 lineage strains. Forty-four (59%) EV-D68-positive patients were children aged ≤ 5 years. Ten patients had severe respiratory or neurological symptoms and one died.

We report an outbreak of enterovirus D68 (EV-D68) infections in Stockholm, Sweden in late August and September of 2016 caused by the newly described B3 lineage [1].

## Patients, samples and routine diagnostics for respiratory viruses

The main study was based on respiratory samples analysed at the Department of Clinical Microbiology, Karolinska University Hospital, Stockholm, Sweden, between 22 August and 25 September 2016 (n = 495; 183 nasopharyngeal aspirates, 232 nasopharyngeal swabs, 77 lower respiratory tract samples, 3 unspecified respiratory samples). The laboratory provides diagnostic services to six of seven major hospitals and approximately half of outpatient care in the Stockholm county (2.2 million inhabitants). Most samples (480 of 495) were from the catchment area and collected as part of routine diagnostics from inpatients and, to a lesser degree, outpatients. Fifteen samples were referred from other counties. For comparison, results from routine EV and rhinovirus diagnostics from the Karolinska University Hospital in 2014, 2015, and the remaining part of 2016 up to 13 November were also analysed.

EV, rhinovirus and 10 other respiratory viruses were diagnosed using in-house real-time polymerase chain reactions (PCR)s [1]. The PCRs for EV and rhinovirus cross-react because the viruses are closely related. Based on results from extensive validation including sequencing, samples with dual reactivity for EV and rhinovirus were classified as rhinovirus if the PCR cycle threshold (Ct)-value for rhinovirus was > 3 lower than the Ct-value for EV and otherwise as EV. Influenza A, influenza B and respiratory syncytial virus were diagnosed using the commercial Simplexa system [2]. The study was approved by Regional Ethical Review board in Stockholm, Sweden (registration number 2016/2004–32).

## Enterovirus D68 polymerase chain reaction and sequencing

A real-time EV-D68 PCR was introduced in the late summer of 2016 and was based on the primers and probe of Piralla et al. [3] and used 5 µL of extracted RNA, 5 µL TaqPath 1-Step RT-qPCR Master Mix, CG (Thermo Fisher Scientific, Stockholm, Sweden), 100 nM of primers and probe in a total volume of 20 µL. An ABI7500 FAST Real Time PCR System (Applied Biosystems, Thermo Fisher Scientific, Stockholm, Sweden) was used with the following cycling profile: 2 min at 25 °C, 15 min at 50 °C, 2 min at 95 °C, and 45 cycles of denaturation for 10 s at 95 °C, annealing for 30 s at 60 °C.

Sequencing of the viral protein (VP)4/VP2 region of the EV/rhinovirus genome was performed with an in-house protocol and primers by Wisdom et al. [4,5]. EV-D68 sequences were deposited in GenBank. The EV/rhinovirus species and type were determined by maximum likelihood phylogenetic trees constructed using Molecular Evolutionary Genetics Analysis (MEGA) 7.0.18 (GTR + I + G model), which included reference sequences available at www.picornaviridae.com and EV-D68 sequences that were downloaded from GenBank after a search using basic local alignment search tool (BLAST).

## Description of the enterovirus D68 outbreak in Stockholm in the early autumn of 2016

**Figure 1:**
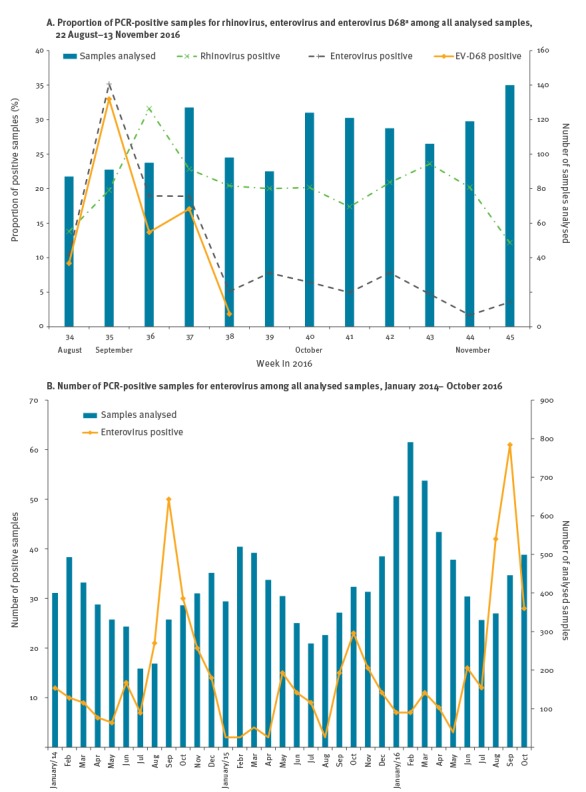
Results of polymerase chain reaction (PCR) analysis of routine respiratory samples, Stockholm, Sweden, 2014–2016

Of the 495 respiratory samples obtained in the main study period between 22 August to 25 September, 72 were positive for rhinovirus alone while 122 (>25%) reacted as EV positive. Among these 122 samples, 21 tested positive for EV alone, and 101 were dually reactive for both rhinovirus and EV. Based on the analysis of Ct-values, 67 of the 101 dually reactive samples most likely contained EV alone, while 34 of these samples likely bore only rhinovirus. Thus a total of 88 samples were classified as EV positive and 106 samples were classified as rhinovirus positive (Figure 1A). The proportion of EV positive samples during the study period in 2016 (18%; 88/495) was significantly higher than the corresponding period in 2015 (2%; 9/366; p<0.001, Fisher exact test) and also higher than in 2014 (15%; 49/321) (Figure 1B).

EVD-68: enterovirus D68.

^a^ Enterovirus D68 data are not available from 25 September onwards, after which the test was only done on demand.

A total of 149 respiratory samples from the study period were analysed with EV-D68 PCR. These included a subset of 34 samples that had tested positive for rhinovirus alone in earlier PCRs, and 115 samples with available material, among the 122 initially appearing as EV positive; 74 samples were positive for EV-D68. In the week of 29 August (week 35), 33% (30/91) of all respiratory samples were positive for EV-D68 (Figure 1A).

EV-D68-negative samples usually had an indication of rhinovirus based on the Ct-value for rhinovirus being > 3 lower than the Ct-value for EV. In 20 of these samples rhinovirus was verified by VP4/VP2 sequencing. The 34 samples that had tested only positive for rhinovirus in prior PCRs were found negative by EV-D68 PCR.

In Figure 1A the two curves depicting the variations with time of the proportions of EV- and EV-D68-positive samples among all respiratory samples analysed during the study period, have almost identical trajectories. This justifies the classification EV and rhinovirus positive samples based on Ct-values. The Figure also indicates that almost all EV infections in the main study period were caused by EV-D68. After 25 September, specific EV-D68 diagnostics were only done on demand of physicians and the proportion of EV-positive samples remained at a lower level up to 13 November, suggesting that EV-D68 activity was likely low in October and early November.

## The outbreak was caused by the new B3 lineage of enterovirus D68

**Figure 2:**
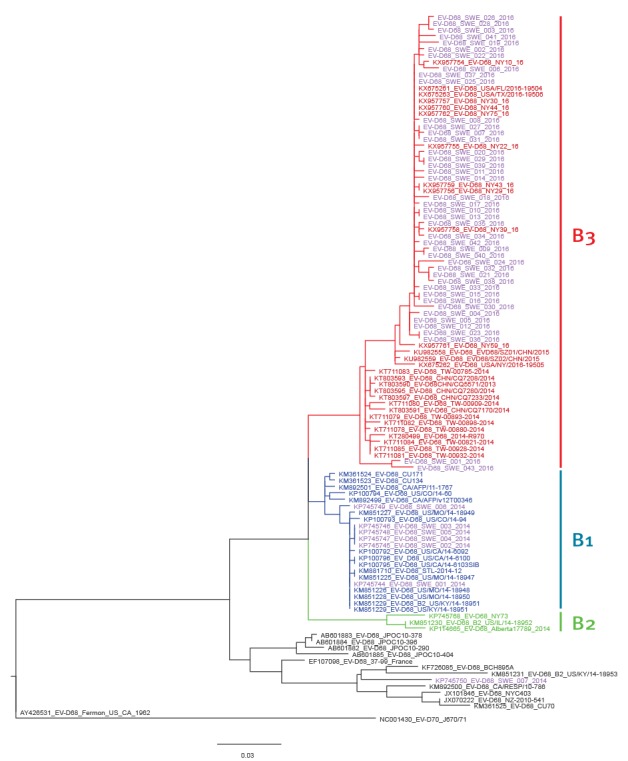
Maximum likelihood phylogenetic tree constructed using enterovirus D68 viral protein (VP)4/VP2 sequences (435 bp) from Stockholm, Sweden and relevant GenBank sequences

VP4/VP2 sequencing was attempted on 80 samples from the study period (57 EV-D68 positive and 23 EV-D68 negative). Figure 2 shows that all successfully sequenced EV-D68 PCR positive samples from 2016 (n = 43) belonged to the recently described B3 lineage of EV-D68 [6]. Within the B3 lineage, 41 of 43 Swedish sequences from 2016 formed a tight cluster together with unpublished Genbank sequences from the United States (US) collected in 2016.

The B1, B2 and B3 lineages of EV-D68 are colour labelled and the new Swedish sequences from 2016 as well as published Swedish sequences from 2014 [4] are purple. The scale bar at the bottom indicates the number of nucleotide substitutions per site, according to the GTR + I + G model. The tree was rooted using the EV-D70 strain J670/71 (NC001430); the branch to the root has been shortened by a factor of ten.

## Characteristics of patients with enterovirus D68 infection

EV-D68 positive patients (n = 74) were significantly older than EV-D68 negative patients (n = 75) (median 3.2 vs 1.1 years, p = 0.039, Mann–Whitney U-test). The proportions of patients in the respective age groups < 1, 1–5, 6–18, and > 18 years were 16% (12/74), 43% (32/74), 22% (16/74), and 19% (14/74), for EV-D68-positive patients, and 37% (28/75), 37% (28/75), 5% (4/75) and 20% (15/75), for EV-D68 negative patients. Female patients accounted for 56% (40/73; for one patient sex was unknown) and 48% (36/75) of the EV-D68 positive and negative samples, respectively. We are aware of ten patients with severe disease diagnosed during the main study period (Table).

**Table:** Characteristics of 10 enterovirus D68 infected patients with severe symptoms, Stockholm, Sweden, 22 August–25 September 2016

Patient code	Age group in years	Sex	Symptoms	Underlying disease	ICU days	Outcome
1	6–18	M	Acute flaccid myelitis Respiratory insufficiency Upper-lower respiratory infection	Previously healthy	17	Not yet fully recovered Still deglutition problem
2	6–18	M	Metabolic crisis Rhabdomyolysis Multiorgan failure	Congenital disorder of metabolism	3	Fatal
3	6–18	M	Respiratory failure	Previously healthy	3	Full recovery
4	1–5	M	Respiratory failure	Asthma	2	Full recovery
5	< 1	M	Respiratory failure	Congenital muscle disease	11	Recovered to original state of health
6	< 1	F	Respiratory failure	Tracheobroncheomalacia	2	Recovered to original state of health
7	< 1	F	Respiratory failure	Congenital chromosomal abnormality	5	Recovered to original state of health
8	> 18	F	Acute flaccid myelitis Bulbar symptoms Upper respiratory infection	Previously healthy	> 6 weeks Ongoing ICU-care	Limited improvement Still paretic
9	> 18	F	Septic Respiratory symptoms Skin rash	Previously healthy	0	Full recovery
10	6–18	M	Acute liver failure Exanthema	Previously healthy	0	Full recovery No other cause of the liver failure has been found Elevated levels of copper in urine in the acute phase to be followed up
11^a^	1–5	F	Acute flaccid myelitis Bulbar symptoms Respiratory insufficiency Upper-lower respiratory infection	Previously healthy	Ongoing ICU-care	Still complete tetraparesis

ICU: intensive care unit.

^a^ Patient diagnosed in October, outside of the main study period from 22 August to 25 September.

## Discussion

In 2014 EV-D68 emerged worldwide [7]. The emergence received high attention by public health authorities because of its magnitude and the clinical presentation of some patients who displayed severe respiratory and neurological symptoms, including acute flaccid paralysis [7-10].

There are indications that EV-D68 may be resurging in 2016 [11,12], but due to lack of systematic surveillance the true disease burden is unclear [7,11]. Here we report a recent large outbreak of EV-D68 infections in Stockholm, Sweden. Severe disease, including one death, was observed in ten of 74 (12%) patients with laboratory-confirmed EV-D68 infection during the study period and one additional patient diagnosed in October 2016. The outbreak peaked in early September and EV activity appears to have been considerably lower in October and early November. It is likely that verified EV-D68 cases represent the tip of an iceberg [7] because patients with milder symptoms are unlikely to have sought medical care or been sampled. Comparison of 2014, 2015, and 2016 indicated that we have documented a true outbreak of EV-D68 in 2016 and that infections also occurred in 2014. This agrees with limited EV-D68 retrospective PCR testing on EV-positive respiratory samples from 2014 (8 EV-D68 positive of 14 samples tested) and 2015 (none EV-D68 positive of 23 samples tested) as well as with published sequencing results on samples from 2014 [4]. It is unlikely that increased awareness and sampling have significantly influenced the findings as the total number of samples received per week was not dramatically different for 2014, 2015 and 2016 and reporting about the 2016 outbreak by the Swedish Public Health Institute and our laboratory to relevant health professionals (mainly paediatricians and infectious disease specialists) only occurred after the peak.

The outbreak was caused by closely related strains of the recently described B3 lineage [6]. Available data indicate that the B3 lineage arose recently in the evolution of EV-D68 and is actively spreading in parts of Europe [12] and the US during the 2016 season (unpublished GenBank sequences). It is unclear if the apparent epidemiological success of this lineage in 2016 is due antigenic drift or if the risk of severe disease differs from other EV-D68s, such as the B1 lineage that caused the worldwide outbreak in 2014.

In a recent rapid risk assessment the European Centre for Disease Prevention and Control (ECDC) stated that the increased numbers of EV-D68 (and EV-A71) detections reinforce the need for vigilance for EV infections, especially cases that present with more severe clinical syndromes [11]. This appears insightful in light of the recent outbreak in Stockholm.
