# Addendum for Euro Surveill. 2016;21(46)

**DOI:** 10.2807/1560-7917.ES.2016.21.47.30409

**Published:** 2016-11-24

**Authors:** Eurosurveillance editorial team

**Affiliations:** 1 European Centre for Disease Prevention and Control, Stockholm, Sweden

In the article entitled ‘Outbreak of enterovirus D68 of the new B3 lineage in Stockholm, Sweden, August to September 2016’ by R Dyrdak et al., published on 17 November 2016, the GenBank accession numbers of the enterovirus D68 sequences were added on 23 November 2016 at the request of the authors.

